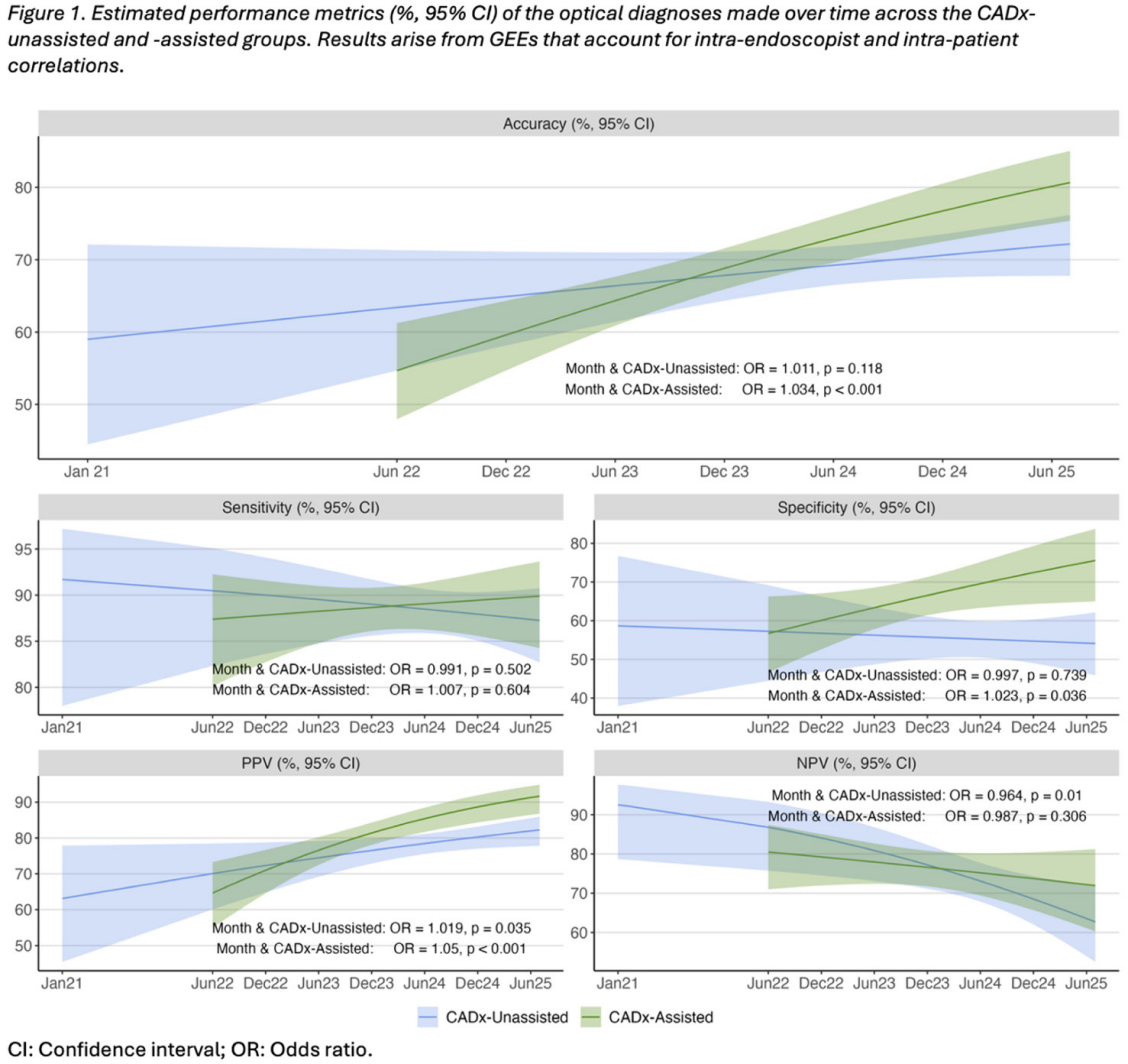# Poster Session I - A66 UPSKILLING OF OPTICAL DIAGNOSIS PERFORMANCE OVER TIME WITH COMPUTER ASSISTED DIAGNOSIS USE: RESULTS FROM A LARGE PROSPECTIVE COHORT

**DOI:** 10.1093/jcag/gwaf042.066

**Published:** 2026-02-13

**Authors:** M Oleksiw, D K Rex, C Hassan, V Michal, R Djinbachian, D von Renteln

**Affiliations:** Centre Hospitalier de l’Universite de Montreal, Montreal, QC, Canada; Indiana University School of Medicine, Indianapolis, IN; IRCCS Humanitas Research Hospital, Rozzano, Lombardia, Italy; Centre Hospitalier de l’Universite de Montreal, Montreal, QC, Canada; Centre Hospitalier de l’Universite de Montreal, Montreal, QC, Canada; Centre Hospitalier de l’Universite de Montreal, Montreal, QC, Canada

## Abstract

**Aims:**

Artificial Intelligence-based Computer-Assisted Diagnosis (CADx) may improve endoscopist optical diagnosis (OD) performance by providing real-time diagnostic feedback to the endoscopist from which they can learn, leading to possible upskilling. However, few studies have assessed the evolution of OD performance, with and without CADx use, over time.

**Methods:**

We conducted a secondary analysis of a large prospective cohort (CER22.013) of patients undergoing elective colonoscopies at our center between January 2021 and July 2025. All polyps with documented OD, either CADx-assisted or unassisted, and available histopathology were included in our analysis. The primary outcome was the monthly evolution of CADx-assisted OD versus unassisted OD overall accuracy, using polyp histopathology diagnosis as ground truth. We hypothesised that over time the accuracy of CADx-assisted ODs would increase at a higher rate than that of CADx-unassisted ODs given the possibility for endoscopist upskilling.

**Results:**

A total of 1321 patients (mean age 65.9, 46.2% female) with 2855 polyps (mean size 7.25mm, 64.4% adenomas, 19.9% hyperplastic, 6.3% sessile serrated, 9.4% other), of which 1257 (44%) CADx-assisted cases and 1598 (56%) CADx-unassisted cases, were included in our analysis. Proportions of histology types and polyp sizes were similar across CADx-assisted and unassisted cases. 30 endoscopists participated in this study, all of whom made at least one CADx-unassisted OD, while 18 (60%) made at least one CADx-assisted OD, 10 of which performed ≥75% of their colonoscopies with CADx. For CADx-assisted cases, the odds of making a correct OD significantly increased by 3.4% each month (OR:1.034, 95% CI:[1.020-1.049], p < 0.001), whilst for unassisted cases, accuracy did not significantly increase over time (1.1% each month, OR:1.011, 95% CI:[0.997-1.025], p = 0.118), **Figure 1**. Similarly, the odds of correctly identifying a non-adenoma as such (specificity) increased by 2.3% each month for CADx-assisted ODs (OR: 1.023, 95% CI:[1.001-1.046], p = 0.036) but not for unassisted ODs, **Table 1**.

**Conclusions:**

In this cohort with CADx-assisted and unassisted OD data spanning four years, OD diagnostic performance increased at a higher rate over time in CADx-assisted cases compared to unassisted cases, supporting that CADx use may enable endoscopist upskilling.

**Funding Agencies:**

CIHRTRIANGLE